# Large-Scale Validation of a Dual Cross-Attention Network for Automated Sleep Staging Using Wearable Photoplethysmography Signals

**DOI:** 10.3390/diagnostics16050802

**Published:** 2026-03-08

**Authors:** Ruochen Li, Yutao He, Yanan Bie, Jiawei Guo, Lichao Wang, Yao Zhao, Jun Zhong, Wei Zhu

**Affiliations:** 1 Division of Life Sciences and Medicine, School of Biomedical Engineering (Suzhou), University of Science and Technology of China, Hefei 230022, China; liruochen@mail.ustc.edu.cn (R.L.); guojw@mail.ustc.edu.cn (J.G.); 2 Suzhou Institute of Biomedical Engineering and Technology, Chinese Academy of Sciences, Suzhou 215011, China; donnasq@163.com; 3 PLA Naval Medical Center, Naval Medical University, Shanghai 200052, China; heyutao@stu.ouc.edu.cn (Y.H.); csnrwlc@163.com (L.W.); zhaoyao0753@163.com (Y.Z.)

**Keywords:** photoplethysmography, sleep staging, sleep disorder screening, non-invasive, deep learning, transfer learning

## Abstract

**Background:** Sleep staging is vital for diagnosing sleep disorders, but the clinical gold standard, polysomnography, is too intrusive for routine home monitoring. While photoplethysmography (PPG) offers a wearable alternative, achieving high diagnostic accuracy remains challenging due to signal noise and individual variability. **Methods:** We developed DCA-Sleep, a deep learning framework using a Dual Cross-Attention (DCA) mechanism to capture long-range temporal dependencies from raw single-channel PPG. To overcome data scarcity, a cross-modality transfer learning strategy was implemented, pre-training the model on six electrocardiogram (ECG) datasets before extensive validation on a combined cohort of 9738 subjects across nine public datasets (including MESA and CFS). **Results:** DCA-Sleep demonstrated superior robustness, achieving an average F1-score of 0.731 and a Cohen’s Kappa of 0.652 on the MESA dataset, significantly outperforming state-of-the-art baselines. The model showed high sensitivity in detecting Wake and Deep Sleep stages, which are critical for clinical assessment. **Conclusions:** This study provides a large-scale validation of a PPG-based staging tool, confirming its reliability as a non-invasive, scalable solution for long-term sleep monitoring and clinical screening.

## 1. Introduction

Sleep disorders, including insomnia and obstructive sleep apnea, represent a major global health challenge, significantly increasing healthcare costs and morbidity related to cardiovascular and metabolic diseases [1,2,3]. Accurate sleep staging—the process of categorizing sleep into distinct physiological phases—is indispensable for precise clinical diagnosis and long-term health management [4]. According to the AASM guidelines, sleep is classified into Wake, Rapid Eye Movement (REM) and non-REM (N1, N2, N3) stages. Wake is characterized by dominant alpha activity, N1 represents transitional sleep, N2 is marked by sleep spindles and K-complexes, N3 corresponds to slow-wave sleep, and REM is defined by rapid eye movements and muscle atonia [5]. Currently, Polysomnography (PSG) remains the clinical gold standard; however, its reliance on multiple intrusive sensors, specialized clinical environments, and manual expert scoring makes it unsuitable for continuous home-based monitoring. This creates a critical “diagnostic gap” where many patients remain undiagnosed due to the discomfort and limited accessibility of traditional PSG [6].

To bridge this gap, wearable Photoplethysmography (PPG) has emerged as a promising non-invasive alternative due to its affordability and integration into consumer devices [7,8]. Despite its potential, PPG-based sleep staging faces significant hurdles: high sensitivity to motion artifacts and lower signal-to-noise ratios compared to electroencephalogram (EEG) signals [9,10]. This limitation makes transitional stages difficult to distinguish, particularly between N1 and N2. To improve robustness and reduce staging ambiguity under wearable sensing constraints, the present study adopts a unified four-class scheme by merging N1 and N2 into Light sleep (L), while retaining Wake (W), Deep sleep (D, corresponding to N3), and REM (R). Most existing automated algorithms struggle with generalization, often failing when applied to diverse clinical populations or hardware platforms. Furthermore, current research is frequently limited by small sample sizes, which undermines the clinical reliability of the proposed models. Therefore, there is an urgent need for a robust diagnostic framework that not only captures complex physiological patterns in PPG but has also been validated across large-scale, heterogeneous cohorts to ensure real-world reliability.

Recent advancements in deep learning, particularly convolutional neural networks (CNNs) and attention mechanisms, have demonstrated significant potential in extracting complex temporal patterns from physiological signals. Previous research has explored various paradigms for PPG-based sleep staging: Sridhar et al. [11] demonstrated the feasibility of five-class staging using heart rate sequences, while Radha et al. [12] employed cross-modality fine-tuning from ECG to PPG data, albeit on a limited cohort of 60 subjects. More recently, Attia et al. [13] developed SleepPPG-Net2 using multi-source domain training across 2574 subjects to improve generalization. However, even state-of-the-art models exhibit inconsistent performance on complex datasets such as the CAP Sleep Database (κ≈0.60), suggesting that current methods still struggle with subtle sleep microstructures and domain-specific noise.

To overcome these limitations, we propose DCA-Sleep, an advanced fully convolutional network (FCN) integrated with a Dual Cross-Attention (DCA) mechanism. Unlike conventional attention models that often overlook subtle physiological fluctuations in noisy PPG data [14], the DCA mechanism is specifically engineered for fine-grained feature selection and the capture of long-range temporal dependencies across diverse sleep cycles. Furthermore, to address the inherent scarcity of high-quality annotated PPG recordings, we implemented a robust cross-modality transfer learning strategy. By leveraging the shared cardiovascular information between ECG and PPG signals, the model acquires a rich physiological prior during pre-training, which improves its generalization performance and robustness under wearable sensing conditions. This framework was rigorously validated on a large-scale cohort of 9738 subjects, supporting its potential applicability in large-scale sleep monitoring scenarios.

The primary objective of this study is to move beyond laboratory-scale experiments and explore the feasibility of wearable cardiovascular-based automated sleep assessment. The main contributions are as follows:

**Development of a Robust DCA-FCN Architecture:** We introduce a novel framework that combines the spatial feature extraction of CNNs with a dual-stream attention mechanism, optimized for the non-stationary nature of raw PPG signals in four-class sleep staging.

**Large-Scale Validation:** To ensure real-world reliability, we conducted a systematic evaluation across nine public datasets involving 9738 subjects. To our knowledge, this represents one of the more extensive validations of PPG-based sleep staging approaches to date, covering diverse demographic and clinical profiles.

**Cross-Modality Generalization Capability:** Through cross-modality transfer learning, DCA-Sleep achieved an average F1-score of 0.731 on the MESA dataset, outperforming existing cardiovascular-based baseline methods and demonstrating promising generalization performance for scalable portable sleep monitoring applications.

## 2. Methods

### 2.1. Model Framework

**Figure 1:**
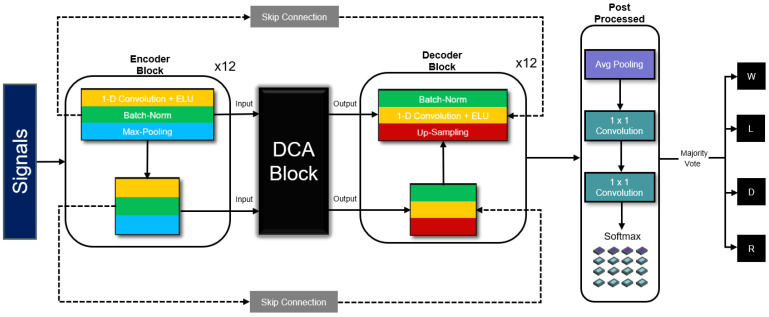
The signal passes through multiple layers of convolutional encoders, dual cross-attention modules, and convolutional decoders in sequence. Each layer of encoders and decoders is connected in a skip manner. Four types of sleep stage predictions are generated through post-processing and majority voting at the end.

Figure 1 depicts the overall structure of the proposed DCA-Sleep model, comprising four principal components: the encoder, the DCA module, the decoder, and the output/post-processing module. Each element is meticulously designed to enable robust feature extraction, context-aware attention, hierarchical reconstruction, and temporal aggregation for accurate sleep stage classification.

Inspired by the U-Net architecture [15] from image segmentation, we adaptively refine it for the sleep staging task. Specifically, our framework integrates a fully convolutional network with dedicated signal pre-processing and post-processing pipelines, and introduces skip connections. This design yields an asymmetric encoder–decoder structure that facilitates multi-scale, multi-view temporal feature extraction and fusion.

At the input stage, a pre-processing module performs signal resampling, outlier clipping, and normalization, allowing the network to accommodate data collected via diverse acquisition modalities, hardware platforms, and filtering protocols, thereby enhancing its generalizability and transferability. At the output stage, high-frequency sleep stage representations capture brief transitions between stages, uncovering microstructural patterns for fine-grained classification. The skip connections [16] resolve feature misalignment between encoder and decoder stages. Notably, our Time-Cross Attention (TCA) mechanism captures global temporal dependencies during multi-layer fusion, while the proposed Channel-Cross Attention (CCA) mechanism addresses inter-channel feature interactions. Together, TCA dynamically highlights salient temporal cues and CCA strengthens cross-channel coherence, adapting to complex sleep dynamics.

The proposed model takes as input 40 consecutive 30 s ECG segments sampled at 128 Hz. Each segment contains 3840 samples, resulting in an input dimension of 153,600 × 1. The final output dimension is 40 × 4, corresponding to four sleep-stage predictions for each 30 s segment. All convolutional layers are one-dimensional with a kernel size of 9 and stride of 1, and temporal downsampling in the encoder is performed using max-pooling with kernel size 2 and stride 2.

The Dual Cross-Attention (DCA) module operates on concatenated encoder–decoder feature maps along the channel dimension and preserves the same temporal resolution after attention-based recalibration. After the final decoding stage, average pooling is applied along the temporal axis to aggregate high-resolution features into fixed-length outputs of size 40 × 4, enabling one prediction per 30 s segment. Finally, to maintain the fully convolutional architecture, a 1 × 1 convolution is employed to linearly map the aggregated multi-channel features into four distinct class logits while preserving the temporal sequence. A subsequent Softmax activation function yields the final sleep stage probabilities for each epoch.

### 2.2. DCA Module

**Figure 2:**
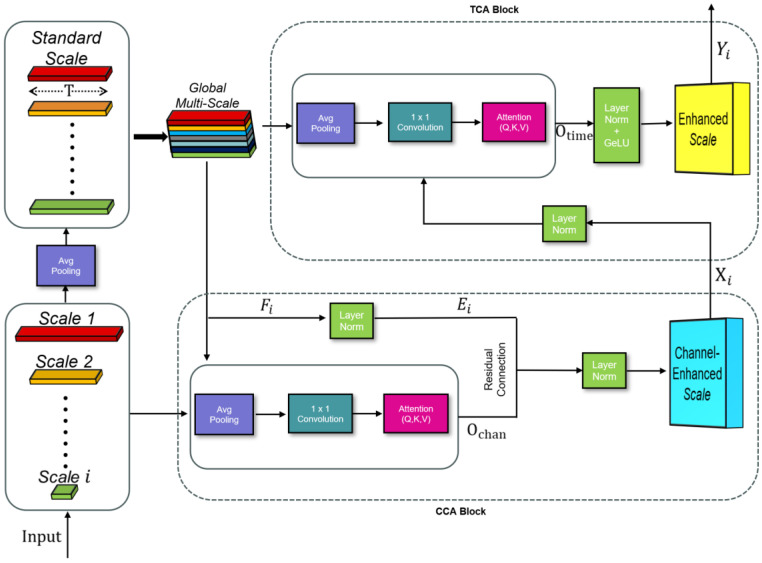
The DCA module sequentially applies CCA to recalibrate channel importance and TCA to capture long-range temporal dependencies, both within residual–normalized blocks to ensure stable training and enriched spatial–temporal feature fusion.

In recent years, the incorporation of attention mechanisms into encoder–decoder architectures have become ubiquitous. A particularly seminal example is the DCA module, originally introduced by Ates et al. [17] for U-Net–based medical image segmentation. The original framework comprised two attention mechanisms: CCA and Spatial Cross-Attention (SCA). Given the temporal nature of our data, we have adaptively refined DCA by substituting the spatial dimension with a temporal axis and enhancing multi-scale feature aggregation. The revised spatial component is rebranded as TCA to more accurately capture and process time-series characteristics. The adapted DCA module framework is illustrated in Figure 2.

To mitigate the impact of signal noise, the DCA module was specifically engineered to automatically identify and reinforce physiological features that are less susceptible to motion artifacts. By performing fine-grained feature selection across both temporal and channel dimensions, this mechanism suppresses non-biological interference while accentuating salient pulse wave morphologies. Consequently, the DCA module enhances the diagnostic robustness of the framework, ensuring reliable sleep stage classification even within the noisy environments typical of wearable PPG monitoring.

Although the model receives a single-channel physiological signal as input, the deep convolutional encoder expands this input into multi-channel feature maps. These channels encapsulate diverse abstract representations, including both critical sleep signatures and unavoidable artifacts. In addition to channel-wise recalibration, the DCA module also incorporates temporal attention to capture long-range dependencies across sleep epochs.

Within the CCA sub-module, the global context engages each per-scale feature map along the channel dimension. A scaled dot-product attention computes inter-channel affinities, dynamically re-weighting channel responses to accentuate physiologically salient signals—such as pulse amplitude modulations—while suppressing redundancies. The CCA-enhanced output is fused with the original representation via residual addition, preserving low-level detail.

The TCA sub-module is specifically designed to capture long-range temporal dependencies and resolve contextual misalignment across different time points, all while mitigating background noise and transient artefacts. For instance, this module effectively highlights abrupt shifts in heart rate variability that correspond to transitions between sleep stages. Sequential application of these modules enriches multi-scale spatial–temporal embeddings, aligns hierarchical features, and fortifies the model’s resilience to noise and domain variations, thereby significantly enhancing the discriminative precision required for four-class sleep staging.

In our implementation of the DCA module, we first apply adaptive average pooling followed by a depthwise separable convolution to each multi-scale encoder feature map $F_{i}\in R^{C_{i}\times T_{i}}$, yielding a fixed-length embedding

(1) $$E_{i}=\mathrm{DConv}1D\left(\mathrm{AP}1D\left(F_{i}\right)\right)\in R^{L\times C_{i}}.$$

These embeddings are then concatenated across the channel dimension to form the global context

(2) $$E_{c}=\mathrm{Concat}\left(E_{1},E_{2},\ldots ,E_{N}\right)\in R^{L\times d_{c}}.$$

Within the CCA sub-module, we project

(3) $$Q_{i}=\mathrm{DConv}1D_{Q}\left(E_{i}\right),\;K_{c}=\mathrm{DConv}1D_{K}\left(E_{c}\right),\;V_{c}=\mathrm{DConv}1D_{V}\left(E_{c}\right),$$

and compute

(4) $$O_{chan}=\mathrm{Softmax}\left(\frac{Q_{i}{K_{c}}^{T}}{\sqrt{d_{c}}}\right)\;V_{c}.$$

The output is refined through residual connection and layer normalization:

(5) $$X_{i}=LayerNorm(O_{chan}+E_{i}).$$

thereby dynamically recalibrating each channel’s contribution. In the TCA sub-module, we reuse the same context to project

(6) $$Q_{t}=\mathrm{DConv}1D_{Q}\left(E_{c}\right),\;K_{t}=\mathrm{DConv}1D_{K}\left(E_{c}\right),\;V_{t}=\mathrm{DConv}1D_{V}\left(E_{c}\right),$$

and perform

(7) $$O_{time}=Softmax\left(\frac{Q_{t}{K_{t}}^{T}}{\sqrt{d_{k}}}\right)V_{t},d_{k}=\frac{d_{c}}{h}.$$

Finally, the temporal output is normalized and activated as

(8) $$Y_{i}=GeLU(LayerNorm(O_{time})).$$

The final DCA-enhanced feature representation is obtained by combining channel- and temporal-enhanced outputs:

(9) $${F_{i}}^{out}=X_{i}+\;Y_{i}.$$

This captures long-range temporal dependencies while filtering transient artifacts.

The research findings indicate that the modified DCA skip connection mechanism is capable of effectively capturing cross-channel and cross-temporal dependencies in time series data, making it well-suited for tasks such as segmentation, classification, or prediction of physiological signals.

### 2.3. Label Processing

**Figure 3:**
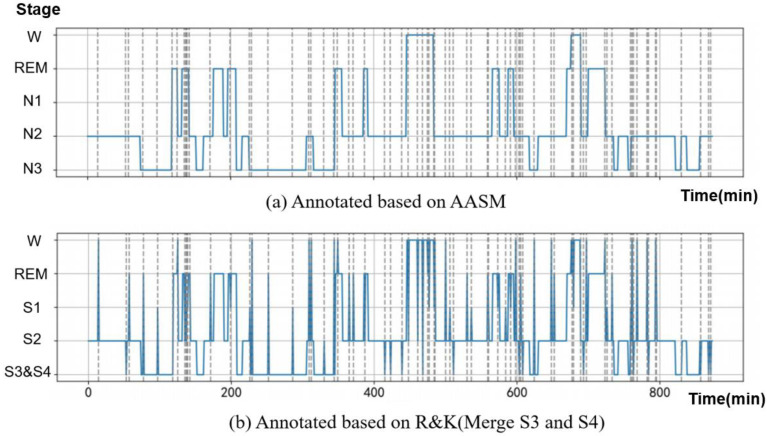
The same sleep data, annotated in accordance with both R&K and AASM standards, exhibits a 45 min discrepancy in scoring. This annotation discrepancy is visually indicated by vertical gray stripes.

According to AASM [5] rules, human sleep is partitioned into Wake, REM sleep, and three progressively deeper non-REM stages (N1–N3). The Rechtschaffen and Kales (R&K) [18] rules, while also including Wake and REM, further subdivide non-REM sleep into four sequential stages (S1–S4), yielding six total stages—one more than under AASM. Figure 3 juxtaposes the staging of the same 45 min segment under both systems, using data from The Dreem Open Datasets (DOD) dataset [19] annotated at 30 s resolution. Panel (a) presents the consensus scoring by five experts following AASM guidelines, whereas panel (b) shows a single rater’s re-scoring according to R&K rules. Discrepancies—highlighted by vertical grey hatching—stem from differences in stage definitions and scoring conventions. Despite these micro-level variations, the macro-scale sleep architecture remains highly congruent, demonstrating that distinct staging frameworks can yield broadly comparable patterns.

To ensure consistency across datasets and align with the intrinsic characteristics of PPG signals, we employ a unified four-class sleep staging: Wake (W); Light Sleep (L), combining N1  +  N2 under AASM and S1  +  S2 under R&K; Deep Sleep (D), corresponding to N3 in AASM and S3  +  S4 in R&K; and REM (R).

### 2.4. Transfer Learning

In the context of wearable physiological monitoring, PPG presents a cost-effective and user-friendly alternative to ECG [9] due to its seamless integration into wrist-worn devices. However, the inherent scarcity of high-quality, clinically annotated PPG datasets (the ‘gold standard’ label scarcity) poses significant challenges for training and optimizing robust deep learning models [12]. While some studies attempt to learn a direct mapping from PPG to ECG signals using paired data, this approach is often undermined by high inter-subject variability and the resource-intensive nature of acquiring high-fidelity paired recordings [20].

To address these limitations, we implemented a two-stage cross-modality transfer learning strategy. This approach leverages the shared cardiovascular regulatory characteristics between ECG and PPG signals as robust physiological priors. By pre-training the DCA-Sleep network on large-scale ECG repositories (encompassing over 10,000 recordings), the model acquires fundamental knowledge of cardiac rhythms and autonomic nervous system dynamics. Subsequently, targeted fine-tuning on limited PPG datasets allows the model to adapt these priors to the specific morphological characteristics of pulse waves. This paradigm effectively bridges the gap between different sensing modalities and significantly improves the model’s generalization across diverse clinical cohorts [13].

### 2.5. Datasets

To comprehensively evaluate the proposed DCA-Sleep framework and its cross-modality transfer learning strategy, this study utilized a diverse collection of publicly available sleep datasets. Depending on their specific roles within our experimental pipeline, these datasets are categorized into two distinct groups: those utilized for initial model pretraining, and those employed for subsequent fine-tuning and evaluation.

#### 2.5.1. Pretraining Datasets

Six datasets were selected for the pre-training and development of our sleep stage classification model, encompassing a total of 7344 subjects and over 10,000 sleep recordings. These datasets represent diverse populations, health conditions, geographical regions, collection periods, and hardware platforms. Detailed characteristics of each dataset are summarized in Table 1, with scoring systems that include both AASM and R&K rules. Notably, The Sleep Heart Health Study (SHHS) dataset [21] requires authorized access, whereas the remaining datasets can be downloaded directly from their official websites. The DOD dataset employs an expert consensus annotation protocol: five independent experts scored the sleep stages, and the final labels were derived through a consensus procedure, effectively minimizing individual bias and error. Consequently, we use DOD as an objective reference standard for rigorous model testing and evaluation.

ECG recordings from all utilized datasets were employed as single-channel model inputs. To reconcile heterogeneous native sampling frequencies, we implemented a preprocessing pipeline that resamples each ECG trace to 128 Hz, removes extreme outliers, and applies global z-score normalization. Sleep stage annotations followed varying standards—The Sleep-EDF Database Expanded (SEDF) [22] and SHHS used the R&K rules; The Danish Center for Sleep Medicine (DCSM) [23], The Physionet Dataset (PHYS) [24], A comprehensive public dataset for sleep researchers (ISRUC) [25] and DOD conformed to AASM rules. Notably, PHYS labels were generated by six independent scorers, and the DOD labels arose from consensus among five experts. We thus conducted a harmonization procedure to align all labels to a consistent four-class sleep staging (see part of “Label Processing”). For model training, 20 min windows (40 consecutive 30 s epochs) were fed into the network to capture long-term temporal dependencies.

#### 2.5.2. Fine-Tuning Datasets

During the fine-tuning phase, three publicly available PPG datasets were employed: The Multi-Ethnic Study of Atherosclerosis (MESA) [26], The Cleveland Family Study (CFS) [27], and The Cyclic Alternating Pattern (CAP) [28]. The MESA dataset initially comprised polysomnography recordings with concurrent PPG signals from 2056 participants, captured at 256 Hz using a Nonin 8000 sensor (Nonin Medical, Inc., Plymouth, MN, USA), of which 2054 were retained after excluding two subjects due to unusable waveforms caused by severe signal corruption. The CFS data were drawn from the fifth follow-up visit of adult participants (aged ≥ 18 years), yielding 320 PPG recordings at 128 Hz, also measured with the Nonin 8000. Four records were excluded due to inadequate signal quality (e.g., excessive motion artifacts or signal dropout), resulting in 316 valid samples. The CAP dataset comprises PPG recordings from 24 subjects—5 diagnosed with insomnia, 8 with nocturnal frontal-lobe epilepsy, 7 with REM behavior disorder, and 4 healthy controls—acquired at 128 Hz for model fine-tuning. Among them, the MESA dataset and the CFS dataset require authorization application. All three datasets employ the R&K rules, and we also align all labels to a consistent four-class sleep staging to ensure annotation consistency and comparability across evaluations. Detailed characteristics of each dataset are summarized in Table 2.

#### 2.5.3. Subject-Based Cross-Validation Protocol

To ensure rigorous and subject-independent model evaluation with robust performance assessment, subject-based cross-validation was adopted for all datasets. Except for the CAP dataset, ten-fold cross-validation was applied. Due to the relatively limited number of subjects in CAP, five-fold cross-validation was used instead.

For datasets using ten-fold cross-validation, subjects were partitioned into ten mutually exclusive folds. In each iteration, one fold was used as the test set, one randomly selected fold was used as the validation set, and the remaining eight folds were used for training. For the CAP dataset, a similar procedure was followed under a five-fold protocol, where one fold was used as the test set, one randomly selected fold was used as the validation set, and the remaining three folds were used for training. This process was repeated until each fold served as the test set once.

This design guarantees subject-level independence across splits while maximizing data utilization for robust model development and evaluation.

### 2.6. Training Strategy and Benchmarking

Due to the inherent characteristics of sleep stages and individual variations, the frequency and duration of each sleep stage may vary considerably [29], potentially leading to a significant class imbalance. To address this issue, we implement a stratified random sampling strategy during the pre-training phase. First, a target stage is uniformly sampled from the label set {W, L, D, R}, ensuring that each class has an equal probability of selection. Once a stage is chosen, a 30 s segment annotated with that stage is randomly drawn from the ECG recordings, guaranteeing that the sample carries a definitive label. This segment is then inserted at a random position within a sliding window of length T=40 epochs (i.e., 20 min), thereby enriching contextual variability. Such random placement simulates the natural variability of sleep stage sequences, enabling the model to learn discriminative features across diverse temporal contexts. Through this three-step procedure—class selection, segment sampling, and window insertion—we balance the representation of all sleep stages in the training data, promoting robust feature learning and enhancing overall generalization performance.

During the training phase, we adopted two complementary paradigms.

The first paradigm involved pre-training on publicly ECG signals from PSG datasets; owing to the high annotation fidelity but limited sample sizes of the DOD and ISRUC cohorts, these datasets were reserved as independent test sets to rigorously assess model generalization.

For comprehensive benchmarking, we compared against five baseline algorithms:

**U-Sleep** [30], a foundational fully convolutional network employing skip connections without attention enhancements;

**DeepSleepNet** [31], a pioneering single-channel architecture integrating convolutional and recurrent neural modules;

**AttnSleep** [32], a time-context encoding model underpinned by multi-head self-attention;

**HierCorrPool** [33], a graph neural network method modeling hierarchical channel correlations within single-channel inputs;

**FC-STGNN** [34], a spatial–temporal graph neural network capturing inter-channel dependencies.

The second paradigm entailed fine-tuning the pre-trained network on PPG data by unfreezing the decoder or epoch classification layers, thereby evaluating the efficacy of cross-modal transfer learning. We also compared the model results using only ECG data and those using only PPG data on the MESA dataset. The details will be introduced in the following experimental results.

## 3. Experimental Results

### 3.1. Model Pre-Training and Evaluation

Our model was initially pre-trained on ECG signals extracted from multiple PSG datasets. Each comparative experiment followed a cross-dataset evaluation protocol, where one dataset was completely held out for testing while the remaining datasets were used for training. Within the training datasets, subject-based 10-fold cross-validation was performed, and performance was reported as the mean ± standard deviation (Mean ± SD) of the F1 score across folds. The aggregated results are presented in Table 3. In Table 3, “D” indicates that the dataset was included in the training set during pre-training, whereas “N” denotes that the dataset was completely held out and used exclusively for testing.

The experimental results indicated, our model achieves consistent four-stage classification performance across six PSG datasets, with overall F1 scores clustered around 0.72 (range: 0.71–0.76). Datasets partially included in pre-training (marked “D”) such as SHHS and PHYS yield the highest scores, whereas fully held-out test datasets (marked “N”) like ISRUC and DOD exhibit slightly reduced performance, indicating reasonable but imperfect generalization.

Stage-specific performance reveals that Wake and REM consistently attain the highest F1 scores. This reflects both the more pronounced ECG signatures of these stages—marked heart-rate variability shifts and distinct waveform morphologies—and a larger volume of training samples collected during wake and REM periods (due to long recording spans and frequent awakenings), which further biases the model. Although random augmentation and window-sampling mitigate imbalance, residual skewness remains.

By contrast, Light Sleep and Deep Sleep are more challenging to distinguish, yielding lower and more variable F1 scores (e.g., Light Sleep: 0.644 on SHHS vs. 0.579 on DOD). The physiological boundaries between light and deep sleep are inherently ambiguous—even expert scoring may differ—so these stages are often merged or post-processed in consumer sleep trackers to improve reliability [35].

Overall, the model maintains an average F1 score of approximately 0.72 across diverse recording conditions, subject populations, and annotation schemes. However, this performance level remains relatively modest, indicating that there is still considerable room to enhance the model’s generalization capabilities.

### 3.2. Baseline Method Comparison

Five benchmark algorithms were selected and evaluated on single-lead ECG recordings from four PSG datasets, including ISRUC, SEDF SC, SEDF ST, and DCSM. In addition, to further investigate modality differences, comparative experiments between ECG and PPG signals were conducted on the MESA dataset. To ensure a fair and rigorous comparison, all baseline models were re-implemented and trained from scratch within our unified experimental framework. While the core network architectures and primary hyperparameters were kept consistent with their original publications, necessary structural adaptations were made to accommodate our specific input dimensions and the adopted four-class sleep staging protocol.

Each experiment was conducted using a subject-based 10-fold cross-validation protocol, and performance was summarized as the mean ± standard deviation of the F1 score (Mean ± SD). Detailed results are presented in Table 4.

Based on the experimental findings, our model consistently achieves the top or second-best performance across all evaluated datasets, outperforming every baseline method, with especially pronounced advantages when evaluated on PPG signals. This underscores its robust modeling capacity for sleep stage classification, as well as its superior generalization and stability. By contrast, HierCorrPool and FC-STGNN exhibit comparatively weaker results on all tasks, likely reflecting either their less suitable modeling paradigms for the current data or a need for more sophisticated preprocessing. AttnSleep delivers the highest F1 score on the SEDF-SC dataset and closely approaches our model’s performance on the remaining cohorts. U-Sleep, while slightly trailing behind AttnSleep and our method, still outperforms DeepSleepNet and significantly surpasses the more recent HierCorrPool and FC-STGNN models, reaffirming that attention mechanisms remain a powerful means to bolster sleep staging accuracy. Notably, DeepSleepNet—despite its earlier design—continues to achieve respectable performance across several datasets, which explains its widespread integration in contemporary sleep monitoring systems.

### 3.3. Transfer Learning and Evaluation

This experiment transfers knowledge learned during ECG pre-training to PPG datasets. We employ three publicly available PPG datasets (MESA, CAP, CFS) to fine-tune and evaluate the ECG-pretrained model, comparing three transfer strategies:

Fine-tuning decoder and segment classifier only $F_{D+S}$;

Fine-tuning decoder, segment classifier, and attention module parameters $F_{D+S+A}$;

Fine-tuning segment classifier only $F_{S}$.

During fine-tuning, layers designated as “frozen” maintain their parameters and weights unchanged. Each experiment was conducted using a subject-based cross-validation protocol. The results are presented in Table 5. The training and validation loss curves of the optimal $F_{D+S}$ model confirm stable convergence without overfitting (see Supplementary Figure S1).

The results compared three transfer learning strategies: fine-tuning the decoder and segment classifier only $F_{D+S}$, fine-tuning decoder, classifier, and attention module $F_{D+S+A}$, and fine-tuning the segment classifier alone $F_{S}$—on the MESA, CAP, and CFS datasets. The $F_{D+S}$ approach consistently yields the highest performance in both F1 scores (0.731, 0.770, 0.747) and Cohen’s kappa (0.652, 0.716, 0.718) across all three cohorts. In contrast, $F_{D+S+A}$ shows a slight drop in performance, and $F_{S}$ exhibits the lowest metrics.

These results indicate that selectively adapting the decoder and classifier strikes the optimal balance between preserving pretrained high-level representations and accommodating modality-specific characteristics of PPG signals. By freezing the bulk of the network (including the encoder and attention parameters), $F_{D+S}$ avoids overfitting and reduces the risk of catastrophic forgetting, while still allowing sufficient plasticity to adjust decision boundaries. Extending fine-tuning to the attention module $F_{D+S+A}$ introduces additional trainable parameters that may overfit small PPG subsets, and restricting adaptation to the classifier alone $F_{S}$ limits the model’s capacity to realign feature distributions, resulting in suboptimal generalization.

**Figure 4:**
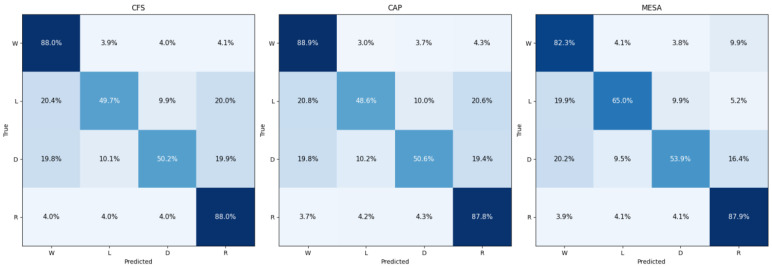
Row-normalized confusion matrices on CFS, CAP and MESA, with per-stage accuracies rounded to one decimal place. The color intensity reflects the performance, with darker shades corresponding to higher accuracy values and lighter shades indicating lower values.

To provide a more granular evaluation, Table 6 and Figure 4 present the confusion matrices and stage-specific performance metrics of the optimal $F_{D+S}$ transfer strategy across the three fine-tuning datasets. The corresponding 95% confidence intervals for stage-specific metrics are provided in Supplementary Figure S2. While the proposed approach achieves competitive overall F1-scores and high sensitivity for Wake and REM stages, consistent performance degradation is observed between Light (L) and Deep (D) sleep. This pattern is consistent across datasets and reflects the intrinsic physiological characteristics captured by single-channel PPG signals. PPG primarily encodes autonomic nervous system dynamics, particularly cardiovascular variability, which exhibits pronounced transitions during Wake and REM but evolves more gradually between Light and Deep sleep. Consequently, these stages present highly overlapping representations in pulse-wave morphology, limiting separability under unimodal sensing. To provide a more comprehensive evaluation under class imbalance conditions, we further report Balanced Accuracy (BA) and Matthews Correlation Coefficient (MCC). These metrics demonstrate that, despite reduced separability between Light and Deep sleep, the framework maintains robust global reliability for macro-architecture sleep assessment. Future work will investigate enhanced temporal modeling strategies to better capture subtle autonomic transitions between these stages.

### 3.4. Strategies Comparison

We assessed transfer learning on the MESA dataset using five approaches—ECG-only training, PPG-only training, and three hybrid transfer-learning strategies. Each experiment was conducted using a subject-based 10-fold cross-validation protocol, and performance was summarized as the mean ± standard deviation of the F1 score (Mean ± SD). As shown in Table 7, using PPG data exclusively for training results in a modest drop in performance, largely attributable to inherent differences in signal properties—such as pulse transit time variability and susceptibility to motion artifacts [36]. However, training exclusively with PPG data still achieves good classification accuracy. These findings underscore that, despite their distinct physiological origins, ECG and PPG signals share salient features that can be leveraged for effective sleep-stage classification.

Moreover, all three transfer-learning methods outperform single-signal training, confirming the feasibility and advantage of transfer learning in this context. By leveraging knowledge learned from ECG, these strategies not only improve adaptability to PPG signals but also reduce the amount of PPG-specific data required for acceptable performance—underscoring their potential to enhance model robustness and data efficiency. Finally, the best results are obtained $F_{D+S}$ by fine-tuning both the decoder and classifier, which aligns perfectly with our conclusions.

## 4. Discussion

This study systematically evaluated the performance and clinical potential of DCA-Sleep, a novel fully convolutional network integrated with a Dual Cross-Attention mechanism, for automated sleep staging using single-channel PPG signals. Validated on an extensive cohort of 9738 subjects, our findings demonstrate that DCA-Sleep offers a robust, non-invasive alternative to traditional PSG-based monitoring.

### 4.1. Performance Analysis

DCA-Sleep achieved consistent four-stage classification performance, with an average F1-score ranging from 0.72 to 0.73 across heterogeneous datasets, representing an improvement of approximately 0.02–0.05 in macro-F1 over established baselines. To contextualize these results within clinical practice, it is important to consider the inherent variability of human sleep staging. Prior studies report inter-rater reliability among experienced sleep technologists ranging from Cohen’s κ ≈ 0.68 to 0.85 under standardized AASM conditions, with overall agreement around 0.76 in multi-center evaluations [5,37]. Given that DCA-Sleep operates under substantially reduced sensing conditions using single-channel PPG, its performance approaches the lower bound of expert agreement under substantially reduced sensing conditions.

Although an error rate approaching 20% may appear substantial in isolation, misclassifications occur primarily between adjacent NREM stages (Light and Deep sleep), a distinction known to exhibit variability even among human scorers. Importantly, the model maintains high sensitivity for Wake and REM stages (Recall > 0.80), which are central to assessing sleep fragmentation and overall sleep–wake dynamics. Therefore, while the proposed framework is not intended to replace full PSG-based diagnostic scoring, it is well suited for macro-architecture analysis and longitudinal population-level monitoring within the physiological constraints of wearable cardiovascular sensing.

### 4.2. Rationale for Excluding EEG-Based Comparison

Although EEG-based PSG remains the clinical gold standard for sleep staging, EEG acquisition typically requires multi-channel scalp electrodes and laboratory-grade instrumentation, representing a fundamentally different monitoring paradigm compared to wearable cardiovascular signal–based approaches. EEG primarily reflects cortical neurophysiological activity, whereas PPG captures peripheral cardiovascular dynamics through blood volume variations.

Given these substantial differences in physiological origin, signal characteristics, and acquisition settings, direct cross-modality performance comparisons would not constitute a methodologically equivalent evaluation framework. Moreover, the primary objective of this study is to develop and validate a wearable, cardiovascular signal–based sleep staging approach suitable for long-term and home-based monitoring, rather than to compete with laboratory-based EEG systems. Therefore, all baseline models were re-implemented and evaluated under identical cardiovascular signal conditions to ensure experimental consistency, fairness, and meaningful comparability of results.

### 4.3. Cross-Modality Transfer

The proposed $F_{D+S}$ transfer strategy effectively leverages shared cardiovascular dynamics between ECG and PPG signals, partially mitigating the challenge of limited annotated PPG data. By selectively fine-tuning the decoder and classifier layers, the model demonstrates stable performance across heterogeneous cohorts. However, as no formal adversarial domain adaptation techniques were incorporated, residual domain discrepancies between ECG and PPG—arising from waveform morphology differences and distinct sensing principles—may persist.

### 4.4. Limitations and Considerations

Despite offering a scalable framework for continuous home monitoring, several limitations warrant explicit acknowledgment.

Physiological constraints and stage ambiguity: Consistent with prior wearable sleep staging studies, distinguishing between Light (L) and Deep (D) sleep remains challenging, with an observed inter-stage misclassification rate of approximately 15–20%. This limitation reflects the inherent boundaries of single-channel cardiovascular sensing. While PPG captures autonomic nervous system dynamics, it lacks access to central nervous system microstructures that are critical for precise NREM differentiation.

Generalizability across clinical populations: Although validated on large-scale cohorts, the datasets may not fully represent severe sleep disorders, complex neurological conditions, extreme age groups, or pronounced cardiovascular comorbidities (e.g., arrhythmias) that directly affect PPG morphology. The robustness of the framework under these specific pathological conditions requires further investigation.

Absence of hardware-level validation: While designed for wearable deployment, DCA-Sleep currently remains an algorithmic proof-of-concept evaluated on high-performance computational platforms. Systematic assessment of memory footprint, energy consumption, and real-time inference latency on consumer-grade devices has not yet been conducted.

### 4.5. Future Work

To facilitate clinical translation and address the aforementioned limitations, several directions merit further investigation. First, multimodal signal integration, such as combining PPG with actigraphy or respiratory effort, may help bridge the specific inter-stage gap between Light and Deep sleep by providing complementary physiological constraints. Second, hardware-aware optimization strategies, including structured pruning and low-bit quantization, are necessary to systematically evaluate real-time edge deployment feasibility on wearable platforms. Third, longitudinal validation across diverse pathological subgroups, together with personalized adaptation to individual physiological baselines, will be essential to further improve generalizability and clinical robustness.

## 5. Conclusions

In this study, we presented DCA-Sleep, a deep learning framework for large-scale automated four-class sleep staging using single-channel PPG signals. Through validation across nine heterogeneous datasets comprising 9738 subjects, the proposed model demonstrated stable performance and effective cross-modality transfer from ECG to PPG signals.

The integration of the Dual Cross-Attention mechanism contributed to improved feature representation under wearable sensing constraints, particularly in the identification of Wake and REM stages. While further clinical validation and hardware-level optimization are required, the proposed approach provides a promising foundation for scalable, non-invasive sleep monitoring and long-term home-based assessment.

## Figures and Tables

**Table 1:** Details of Pretraining Datasets (The SHHS dataset requires authorized access).

Dataset	Subjects	Records	Gender Ratio (M:F)	Avg Age (Years)	Total Duration	Scoring System
DCSM	255	255	N/A	N/A	~200 days	AASM
PHYS	994	994	2:1	55.2	~310 days	AASM
SEDF-SC/ST	100	197	1:1.5	54.7	~156 days	R&K
ISRUC	118	126	2.75:1	49.8	~40 days	AASM
SHHS	5797	8444	1:1	63.1	~3144 days	R&K
DOD	80	80	2.3:1	42.4	~27 days	AASM

**Table 2:** Details of Fine-tuning Datasets.

Dataset	Subjects	Records	Gender Ratio (M:F)	Avg Age (Years)	Total Duration	Scoring System
MESA	2054	2054	1:1.2	69.4	~900 days	R&K
CFS	316	316	1:1	55.2	~8 days	R&K
CAP	24	24	1:1.2	41.7	~300 days	R&K

**Table 3:** Pre-training performance on six PSG datasets using single-lead ECG. F1 scores are reported as mean ± standard deviation across subject-based cross-validation folds within the training datasets.

Dataset	Mark	Wake	Light	Deep	REM	F1 Score
DCSM	D	0.803 ± 0.012	0.587 ± 0.007	0.691 ± 0.011	0.859 ± 0.009	0.73
PHYS	D	0.809 ± 0.009	0.621 ± 0.004	0.642 ± 0.012	0.908 ± 0.010	0.75
SEDF-SC/ST	D	0.822 ± 0.008	0.608 ± 0.021	0.613 ± 0.016	0.877 ± 0.005	0.71
ISRUC	N	0.795 ± 0.007	0.581 ± 0.003	0.613 ± 0.005	0.799 ± 0.011	0.71
SHHS	D	0.880 ± 0.019	0.644 ± 0.033	0.691 ± 0.026	0.829 ± 0.002	0.76
DOD	N	0.771 ± 0.013	0.579 ± 0.019	0.704 ± 0.032	0.826 ± 0.004	0.72

**Table 4:** Baseline methods comparison on different datasets. F1 scores are reported as mean ± standard deviation over 10-fold cross-validation; the “Year” column denotes each method’s original publication year. Bold values indicate the best performance.

Dataset	Year	ISRUC (ECG)	SEDF-SC (ECG)	SEDF-ST (ECG)	DCSM (ECG)	MESA (ECG)	MESA (PPG)
DeepSleepNet	2017	0.668 ± 0.013	0.673 ± 0.026	0.661 ± 0.017	0.693 ± 0.004	0.622 ± 0.012	0.528 ± 0.024
U-sleep	2021	0.703 ± 0.027	0.711 ± 0.009	0.698 ± 0.008	0.719 ± 0.014	0.657 ± 0.005	0.517 ± 0.018
AttnSleep	2021	0.709 ± 0.012	**0.737 ± 0.022**	0.704 ± 0.025	0.726 ± 0.017	**0.685 ± 0.009**	0.534 ± 0.015
HierCorrPool	2023	0.625 ± 0.042	0.661 ± 0.011	0.646 ± 0.021	0.680 ± 0.016	0.651 ± 0.013	0.495 ± 0.024
FC-STGNN	2024	0.570 ± 0.014	0.655 ± 0.017	0.562 ± 0.011	0.667 ± 0.023	0.604 ± 0.019	0.556 ± 0.021
DCA-Sleep (Our)	2025	**0.714 ± 0.006**	0.729 ± 0.020	**0.709 ± 0.014**	**0.735 ± 0.008**	0.661 ± 0.012	**0.639 ± 0.016**

**Table 5:** Transfer learning strategy performance on PPG datasets. F1 scores and Cohen’s kappa coefficients are reported as mean ± standard deviation over the respective cross-validation folds (10-fold for MESA/CFS, 5-fold for CAP). Bold values indicate the best performance.

Strategy	F1 Score			Cohen’s Kappa		
	MESA	CAP	CFS	MESA	CAP	CFS
$F_{D+S}$	**0.731 ± 0.013**	**0.770 ± 0.025**	**0.747 ± 0.042**	**0.652 ± 0.022**	**0.716 ± 0.006**	**0.718 ± 0.011**
$F_{D+S+A}$	0.726 ± 0.020	0.751 ± 0.016	0.724 ± 0.021	0.647 ± 0.040	0.701 ± 0.015	0.694 ± 0.014
$F_{S}$	0.699 ± 0.017	0.722 ± 0.008	0.708 ± 0.013	0.622 ± 0.025	0.686 ± 0.041	0.681 ± 0.003

**Table 6:** Stage-specific classification performance and global reliability metrics of the optimal $F_{D+S}$ transfer strategy across the three fine-tuning datasets.

Dataset	W		L		D		R		BA	MCC
	Precision	Recall	Precision	Recall	Precision	Recall	Precision	Recall		
MESA	0.858 ± 0.015	0.823 ± 0.012	0.602 ± 0.016	0.650 ± 0.021	0.571 ± 0.014	0.539 ± 0.018	0.892 ± 0.013	0.879 ± 0.010	0.723	0.659
CFS	0.872 ± 0.020	0.880 ± 0.018	0.585 ± 0.024	0.497 ± 0.022	0.594 ± 0.016	0.502 ± 0.029	0.884 ± 0.018	0.880 ± 0.017	0.690	0.731
CAP	0.837 ± 0.035	0.889 ± 0.031	0.551 ± 0.044	0.486 ± 0.041	0.573 ± 0.039	0.506 ± 0.038	0.861 ± 0.030	0.878 ± 0.028	0.690	0.718

**Table 7:** Transfer learning performance on the MESA dataset. F1 scores and Cohen’s kappa coefficients are reported as mean ± standard deviation over the 10-fold cross-validation. “ECG-only” and “PPG-only” denote single-signal baselines, while $F_{D+S}$, $F_{D+S+A}$ and $F_{D}$ represent the three hybrid fine-tuning strategies. Bold values indicate the best performance.

Strategy	F1 Score	Cohen’s Kappa
ECG-only training	0.661 ± 0.012	0.586 ± 0.010
PPG-only training	0.639 ± 0.016	0.534 ± 0.017
$F_{D+S}$	**0.731 ± 0.013**	**0.652 ± 0.022**
$F_{D+S+A}$	0.726 ± 0.020	0.647 ± 0.040
$F_{D}$	0.699 ± 0.017	0.622 ± 0.025

## Data Availability

All datasets used in this study are public or accessible upon application to the custodians. Pre-training used six PSG datasets (DCSM, PHYS, SEDFSC/ST, ISRUC, SHHS, DOD), and fine-tuning/evaluation used three PPG datasets (MESA, CFS, CAP). SHHS, MESA, and CFS require approved data-use access; others are downloadable from their official portals. All records are de-identified. Labels were harmonized to a four-class scheme (Wake/Light/Deep/REM), and signals were preprocessed (resampling, outlier clipping, normalization) as detailed in Methods. We employed independent held-out and cross-dataset testing with repeated runs to report mean ± SD, supporting robustness and reproducibility.

## Supplementary Materials

The following supporting information can be downloaded at: https://www.mdpi.com/article/10.3390/diagnostics16050802/s1, Supplementary Figure S1: Training and validation curves for the proposed DCA-Sleep model using the $F_{D+S}$ transfer strategy. The red dashed line represents the transfer point from ECG pre-training to PPG fine-tuning. The stable descent of the validation loss and the consistent rise in accuracy and recall after the transfer point confirm robust model convergence without overfitting. Supplementary Figure S2: Stage-specific Precision and Recall with 95% confidence intervals across the MESA (N = 10 folds), CFS (N = 10 folds), and CAP (N = 5 folds) datasets. Blue and orange bars represent the mean Precision and Recall, respectively, evaluated using the F_{D+S} transfer strategy. Error bars indicate the 95% confidence intervals across the cross-validation folds, demonstrating the model's stage-level reliability and variance.
