# Supplementary material for: Large-Scale Validation of a Dual Cross-Attention Network for Automated Sleep Staging Using Wearable Photoplethysmography Signals
Source: Diagnostics (Basel). 2026 Mar 8;16(5):802. doi: 10.3390/diagnostics16050802 (PMC12984516; doi:10.3390/diagnostics16050802)

Supplementary Figure S1 Training and validation curves for the proposed DCA-Sleep model using the F_{D+S} transfer strategy. The red dashed line represents the transfer point from ECG pre-training to PPG fine-tuning. The stable descent of the validation loss and the consistent rise in accuracy and recall after the transfer point confirm robust model convergence without overfitting.

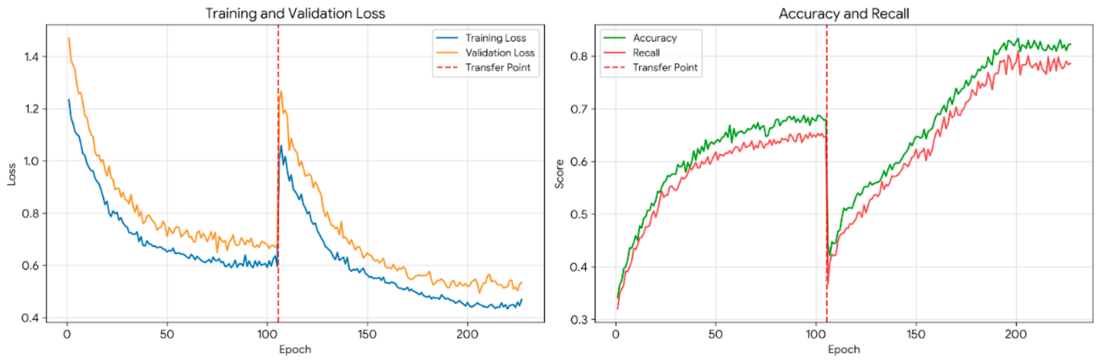

Supplementary Figure S2 Stage-specific Precision and Recall with 95% confidence intervals across the MESA (N=10 folds), CFS (N=10 folds), and CAP (N=5 folds) datasets. Blue and orange bars represent the mean Precision and Recall, respectively, evaluated using the F_{D+S} transfer strategy. Error bars indicate the 95% confidence intervals across the cross-validation folds, demonstrating the model's stage-level reliability and variance.

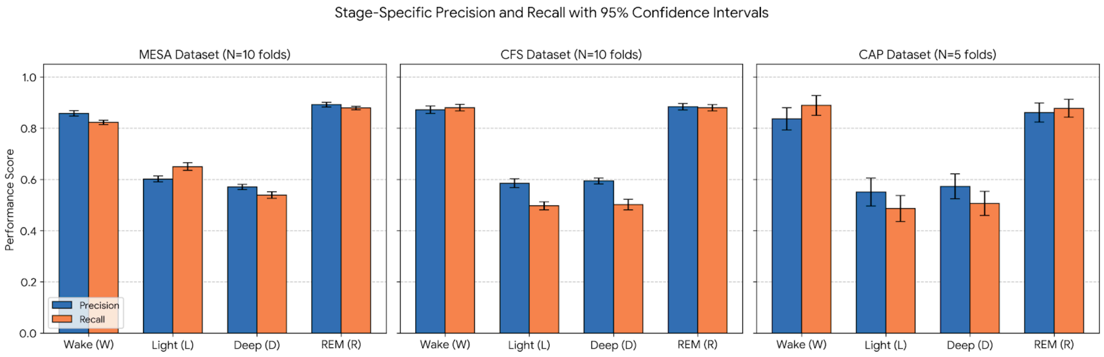

Supplement: Supplementary file 1 [file diagnostics-16-00802-s001.zip › diagnostics-4099740-supplementary.pdf]
